# *In vitro* toxicological evaluation of pouched portioned oral nicotine products

**DOI:** 10.3389/ftox.2024.1452274

**Published:** 2024-11-28

**Authors:** Brian M. Keyser, Robert Leverette, Reagan McRae, John Wertman, Tom Shutsky, Ken Szeliga, Patrudu Makena, Kristen G. Jordan

**Affiliations:** RAI Services Company, Winston-Salem, NC, United States

**Keywords:** modern oral nicotine pouches, *in vitro* toxicity assays, mutagenicity, genotoxicity, cytotoxicity

## Abstract

**Introduction:**

Modern oral nicotine pouch products (ONPs) are a category of oral nicotine products which contain pharmaceutical-grade nicotine, flavors, and other food-grade ingredients but no tobacco leaf. Recent reports indicate that ONPs in general do not contain (or only at minimal levels) the harmful and potentially harmful constituents (HPHCs) identified in cigarette smoke, suggesting their potential as alternative products for reducing harm from cigarette smoking.

**Methods:**

We assessed *in vitro* toxicological effects of eight ONPs, designated as modern oral (MO) 1 to 8 along with an ONP, an oral tobacco (snus), and a combustible cigarette market comparator using established regulatory toxicological assays including Ames, *in vitro* micronucleus (ivMN), and neutral red uptake (NRU) assays.

**Results:**

The MO test products 1-7 ZYN wintergreen, and General Snus white mint were negative for mutagenicity (Ames assay), genotoxicity (ivMN), and cytotoxicity (NRU). The combustible cigarette was positive in all three assays. The MO-8 test product was negative for mutagenicity; however, it was positive in the ivMN and NRU assays at concentrations either 42 to 135-fold based on the ivMN i to iv treatment schedule or 60-fold higher, respectively, when compared to combustible cigarettes.

**Discussion:**

Thus, the MO test products are likely to be less harmful than combustible cigarettes and are alternatives to cigarettes. However, understanding of long-term effects of ONPs in general requires additional research.

## 1 Introduction

A relative exposure risk continuum has been recognized among different tobacco and nicotine-containing products. At the higher end of the risk spectrum are cigarettes, while pharmaceutical products containing nicotine are placed at the lower end with minimal risk (Abrams et al., 2018; Nutt et al., 2014; Institute of Medicine Committee to Assess the Science Base for Tobacco Harm Reduction et al., 2001). The FDA has established a list of harmful and potentially harmful constituents (HPHCs) present in cigarette smoke and identified them as causative agents of several serious smoking-related diseases (Food and Drug Administration, 2012a). Induction of genotoxicity, mutagenicity, and cytotoxicity are among the key mechanisms through which cigarette smoke causes the adverse biological and toxic effects that lead to smoking-related disease (Centers for Disease Control and Prevention et al., 2010a). Although nicotine is addictive and listed as one of the HPHCs, it is recognized as less harmful than the other HPHCs and is not associated with the risk of smoking-related diseases (Food and Drug Administration, 2022; Gottlieb and Zeller, 2017).

As the name implies, smokeless tobacco (ST) products do not generate combustion-related toxicants and are predominantly consumed orally. Although STs contain some HPHCs, epidemiological studies show that US smokeless tobacco products and Swedish snus are significantly less harmful than cigarettes (Henley et al., 2007; Henley et al., 2005; Luo et al., 2007). Swedish snus has also been notably less active in *in vitro* toxicological assays than cigarettes (Coggins et al., 2012). Some varieties of Swedish snus and Copenhagen moist snuff have been authorized for marketing as modified-risk products by the US FDA (Food and Drug Administration, 2019).

Oral nicotine products that contain pharmaceutical-grade nicotine and no tobacco leaf are a relatively new category of smokeless tobacco products with an increasing market presence in the United States and around the world (Campaign for Tobacco-Free Kids, 2024; Jablonski et al., 2022). Some available brands of oral nicotine pouch products (ONPs) in the United States include on!^®^, ZYN, Velo, and Rogue (Campaign for Tobacco-Free Kids, 2024; Ling et al., 2023; Majmundar et al., 2022). These products generally contain pharmaceutical-grade nicotine (tobacco-derived or synthetic), food-grade flavorings, additives, and fillers (e.g., modified cellulose), which are portioned into pouches. These products are considered tobacco products and are under the purview of the FDA Center for Tobacco Products (FDA-CTP). These modern oral products differ from traditional smokeless tobacco products, such as moist snuff or snus, in that they contain minimal to no tobacco-specific nitrosamines and other HPHCs (Azzopardi et al., 2022; Back et al., 2023; Mallock et al., 2024). Hence, ONPs are anticipated to pose a lower risk of exposure to these harmful constituents than cigarettes and traditional STs (Grandolfo et al., 2024; Jackson et al., 2023).

Tobacco harm reduction (THR) is an overall approach to reduce harm from cigarette smoking. THR is about educating adult smokers who are uninterested in quitting about alternatives to combustible cigarettes (Hatsukami and Carroll, 2020; Institute of Medicine Committee to Assess the Science Base for Tobacco Harm Reduction et al., 2001; Zeller et al., 2009). Although ONPs could impart lower risk for smokers who switch to them rather than smoking, their appropriateness for the protection of public health (APPH) must be demonstrated and authorized by the FDA under the Premarket Tobacco Application (PMTA) process (United States Congress, 2009).

In the PMTA rule, the FDA has stated that *in vitro* toxicology studies are a key element of the overall assessments of candidate tobacco products seeking marketing authorization (Food and Drug Administration, 2021). *In vitro* toxicological assays for genotoxicity, mutagenesis, and cytotoxicity have been extensively used to characterize the effects of exposure to various types of tobacco products (Johnson et al., 2009; Lauterstein et al., 2020). The non-clinical studies offer insight into the mechanisms of disease incidence caused by a tobacco product and, more generally, provide context for the data obtained from human studies regarding health risks (Food and Drug Administration, 2021). Assessment of mutagenicity by Ames assay, genotoxicity by *in vitro* micronucleus (ivMN) assay, and cytotoxicity by neutral red uptake (NRU) are three widely used methods for regulatory assessments of tobacco products. Numerous studies have demonstrated that preparations of cigarette smoke induce mutagenic, genotoxic, and cytotoxic responses in these assays (Johnson et al., 2009; Centers for Disease Control and Prevention et al., 2010b).

In this manuscript, we have investigated the *in vitro* toxicological effects of eight modern oral nicotine pouch products that varied in nicotine strength and flavorings. As comparator products, we tested market-leading products from the following categories: combustible cigarettes, snus, and ONPs. The mutagenicity (bacterial reverse mutagenesis or Ames assay), genotoxicity [*in vitro* micronucleus (ivMN) assay], and cytotoxicity [neutral red assay (NRU)] of all study products were evaluated in three well-established regulatory toxicological assays.

## 2 Materials and methods

### 2.1 Study products

The eight ONP variants manufactured by R.J. Reynolds Tobacco Company assessed in this study are not commercially available. These products are designated as MO-1 through MO-8, and the flavor descriptors from a previously published flavor wheel for e-liquids for each are listed in Table 1 (Krüsemann et al., 2019). These MO products are manufactured using tobacco-derived, pharmaceutical-grade nicotine (rather than tobacco leaf) in a cellulose-based matrix with other ingredients for stability and specific to the flavor concentrate of the product, portioned by weight into the pouch using a porous material referred to as the “fleece.” The pouch weight for MO-1 ONP was 400 mg, and the remaining MO-2 through MO-8 ONPs pouches (each) weighed 600 mg (Table 1).

**TABLE 1:** Overview of investigational products.

Test product	Flavor descriptor^a^	Nicotine (mg) per pouch	Pouch weight (mg)	Nicotine (mg/g or mg/cig)^b^
MO-1	Menthol mint	8	400	22.74 ± 1.91
MO-2	Menthol mint	8	600	14.60 ± 1.91
MO-3	Menthol mint	12	600	22.10 ± 2.26
MO-4	Mint	8	600	14.27 ± 1.19
MO-5	Tobacco	8	600	14.69 ± 1.30
MO-6	Tobacco	8	600	14.42 ± 0.83
MO-7	Fruit	8	600	13.99 ± 0.48
MO-8	Spices	8	600	14.58 ± 1.69
ZYN Wintergreen	Wintergreen	6	400	13.90 ± 1.54
General snus white mint	Mint	8	700	6.34 ± 0.81
Marlboro Gold	Non-menthol	N/A	N/A	1.68 ± 0.12

^a^ As described in Krüsemann et al. (2019).

^b^ Average ± standard deviation across all studies reported, CAS, extraction, or DMSO (combustible only) extraction.

N/A: not applicable.

Three different commercially available comparator products were also assessed in parallel with the MO study products (Table 1), which are as follows: ZYN Wintergreen 6 mg nicotine ONP (pouch weight of 400 mg), General Snus White Mint 8 mg nicotine product (pouch weight of 700 mg), and Marlboro Gold cigarettes (non-menthol).

### 2.2 Sample preparation

#### 2.2.1 ONPs and snus

The eight MO test products and the market ONP and snus comparators were extracted in artificial saliva with enzymes [complete artificial saliva (CAS)]. CAS and extracts of all test articles (except the combustible cigarette) were prepared and stored as described previously (Keyser et al., 2024; Keyser, 2022). The resulting CAS extracts were tested for sterility and analyzed for nicotine using the Health Canada method T-301 (Health Canada, 2018b). Nicotine content in CAS extracts was used to express the nicotine concentration, as mg/mL nicotine equivalents were applied to the *in vitro* test systems (Keyser et al., 2024).

#### 2.2.2 Combustible cigarette

Combustible cigarettes had the standard butt length marked according to the International Organization for Standardization (ISO) (International Standards Organization, 2019). Cigarettes were conditioned and smoked per ISO guideline 3,402:1999 on a rotary smoking machine [Körber Technologies Instruments GmbH, Hamburg, Germany (formerly Borgwaldt KC GmbH)] using the Health Canada Intense (HCI) smoking regimen (55 mL puff volume, 2 s puff duration, 30 s puff interval, 100% vent blocking) (International Standards Organization, 2018). Mainstream smoke from multiple cigarettes was passed through pre-weighed 92 mm Cambridge filter pads (Hauni, Richmond Inc., Richmond, VA, United States) to collect a minimum of 180 mg of total particulate matter (TPM) per pad. Pads were extracted in dimethyl sulfoxide (DMSO) to a stock concentration of either 10 mg TPM/mL (Ames, NRU) or 20 mg TPM/mL (ivMN). The resulting TPM extracts were tested for sterility and analyzed for nicotine (Health Canada, 2017a; Health Canada, 2018b). The TPM extracts were either aliquoted and frozen at −70°C until tested (Ames, ivMN) (Crooks et al., 2013) or tested within 1 h of completion of sample generation (NRU) (Health Canada, 2018a **)**. Nicotine content in the TPM was used to express concentration as mg/mL nicotine equivalents applied to the *in vitro* test systems.

### 2.3 *In vitro* assays

A summary of *in vitro* assays employed is presented in Table 2. All *in vitro* assays were conducted in accordance with the Organisation for Economic Co-Operation and Development (OECD) principles of Good Laboratory Practice (OECD, 2004; OECD, 2018; Food and Drug Administration, 1999) for each assay type, with three replicate experiments using CAS and TPM samples from three independent test sample preparations.

**TABLE 2:** Summary of biological assays.

Endpoint	Technique	Cell/bacterial system	Metabolic activation	Guideline/protocol/reference
Mutagenicity	Bacterial reverse mutation (Ames) assay	*Salmonella typhimurium* strains TA98, TA100, TA102, TA1535, and TA1537	± S9	OECD TG 471 (2020) Health Canada T-501 (2017)
Genotoxicity	*In vitro* micronucleus assay	Chinese hamster ovary (CHO-WBL) cells	± S9	OECD TG 487 (2016) Health Canada T-503 (2017)
Cytotoxicity	Neutral red uptake assay	CHO-WBL cells	Not applicable	Health Canada T-502 (2017) OECD Guidance Document No. 129 (2010)

#### 2.3.1 Bacterial reverse mutation (Ames) assay

The Ames assay was performed in five strains of *Salmonella typhimurium* (TA98, TA100, TA102, TA1535, TA1537, Molecular Toxicology Inc., Boone, NC, United States) using the pre-incubation method in the absence and presence of an exogenous metabolic activation system (phenobarbital 5-6 benzoflavone-induced rat liver post-mitochondrial supernatant S9, 5%) according to OECD guideline No. 471 (OECD, 2020) and Health Canada Test Method T-501. The MO test products and market ONP and snus comparators were tested at doses from 0 to 15 mg smokeless product/plate, while combustible cigarettes were tested at doses from 0 to 0.5 mg TPM/plate (concentration based on CAS and TPM stock preparations of 300 mg/mL and 10 mg/mL respectively). In each experiment, eight doses of the study sample were tested in triplicate plates. Several known genotoxic chemicals (2-aminoanthracene, benzo [a]pyrene, 2-nitrofluorene, sodium azide, mitomycin C, and 9-aminoanthracene) were used as positive controls. The criteria for a positive mutagenic response were as follows: (i) a concentration-related increase in revertant (spontaneous) colony (a group of bacteria derived from the same mother cell) count; (ii) a statistically significant increase (Dunnett’s test, α = 0.01) in mean revertant colonies/plate over vehicle control, and (iii) a revertant colony count higher than the historical background at the testing laboratory.

#### 2.3.2 *In vitro* micronucleus (ivMN) assay

The *in vitro* micronucleus (ivMN) assay was performed as a complementary genotoxicity test for the Ames assay. The ivMN assay detects micronuclei, small DNA content encapsulated by a nuclear envelope and separated from the primary nucleus (genotoxicity) that are formed due to exposure to a compound during interphase that precedes mitosis or cell division. Phenobarbital 5-6 benzoflavone-induced rat liver post-mitochondrial supernatant (S9) was obtained from Molecular Toxicology Inc. (Moltox^®^; Boone, NC, United States). Extracts of study products were tested per OECD guideline No. 487 and Health Canada Test Method T-503 (OECD, 2016; Health Canada, 2017b) using Chinese hamster ovary (CHO-WBL) cells (Sigma-Aldrich Canada Co., Oakville, ON Canada) in four treatment schedules (without cytochalasin B): (i) short term (3 h) treatment in the absence of S9 followed by recovery (21 h), (ii) short term (3 h) treatment in the presence of S9 followed by recovery (21 h), (iii) long-term (24 h) treatment in the absence of S9 and (iv) extended treatment in the absence of S9 (24 h treatment with 24 h recovery). The use of schedule iv is not described in the OECD guideline. However, based on work examining the sensitivity of the ivMN assay with tobacco products (Thorne et al., 2019), it was included in this study. In each experiment, five doses of the test sample were treated in duplicate flasks. The top concentrations tested in each treatment schedule are shown in Table 3. The following criteria were used to determine if a test sample elicited a positive response: (i) a concentration-dependent increase in the number of MN/2000 cells scored; (ii) a statistically significant (Dunnett’s test, α = 0.01) in the mean frequency of MN for at least one concentration over the vehicle control, and (iii) an increase in the number of MN over the historical background values at the testing laboratory.

**TABLE 3:** ivMN assay: top concentration tested.

Treatment schedule	Top concentration tested	
	MO test products^a^; market ONP and snus comparators (mg SP^b^/mL)	Comparator cigarette (mg TPM/mL)
Schedule (i)	30	0.200
Schedule (ii)	30	0.200
Schedule (iii)	30	0.150
Schedule (iv)	30	0.125

^a^For MO-8, top concentrations under schedules i-iv were 15 mg, 24 mg, 15 mg, and 6 mg smokeless product/mL, respectively.

^b^SP, smokeless product, dose based on the mass of product per mL in CAS extracts.

#### 2.3.3 Neutral red uptake (NRU) assay

Extracts of investigational products were tested using the NRU assay in CHO-WBL cells in the absence of an exogenous metabolic activation system to evaluate the potential to induce cytotoxicity, as described previously (Putnam et al., 2002). In brief, testing was performed in accordance with OECD guideline No. 129 and Health Canada Test Method T-502 (Health Canada, 2017a; OECD, 2010). For the NRU assay, cells were seeded in 96-well microtiter plates at a density of 1 × 10^5^ cells/mL in growth medium and cultured for 24 h. For each study sample, eight doses were tested in quadruplicate. At the end of treatment, the cell culture medium was removed, and the cells were stained with neutral red dye and processed per the HCI method (Health Canada, 2018a). The criterion for a positive response was when an inhibitory concentration 50% (IC_50_), that is, the treatment dose that reduces relative absorbance to 50% of that of vehicle control, could be calculated.

### 2.4 Statistical methods

All statistical methods were performed using SAS analytical software (SAS Institute, Inc., Cary, NC, United States).

#### 2.4.1 Ames and ivMN assays

For each investigational product deemed either mutagenic or genotoxic in a strain or treatment schedule, respectively, the slope was determined for each experiment on a nicotine equivalents dose basis using a Poisson regression model (generalized linear model with Poisson distribution and identity link function) predicting the number of revertants/plate or number of micronuclei in 2,000 scored cells from the test sample dose. In the case of the ivMN assay, only doses with ≤60% toxicity were considered for model fitting. Mean slopes from the three experiments were compared using analysis of variance, followed by *post hoc* paired comparison of the cigarette comparator to each MO product or the snus comparator. *p*-values for comparisons were adjusted using the Bonferroni method to control the family-wise error rate at 0.05. No statistical comparisons were made if an MO test article was deemed negative in the respective assays.

#### 2.4.2 NRU assay

Relative absorbance (%) was calculated on an individual experimental plate basis. Negative corrected absorbance values were adjusted to zero to determine relative absorbance. For each investigational product deemed to be cytotoxic, the IC_50_ was determined for each experiment by fitting the following non-linear sigmoidal model to the dose-response curve and solving for the concentration yielding a 50% reduction (i.e., relative absorbance of 50%) in which the sigmoidal model with the top parameter was fixed at 100 and bottom parameter was fixed at 0:

$$\%\text{ Relative Absorbance}=\frac{a}{{1+10}^{\left[(\log\left(c\right)-\log\left(concentration\right))\times b\right]}}$$

Here,

parameter a represents the maximum value;

parameter b is a “slope parameter” related to the steepness of the curve;

parameter c (=EC_50_ = effective concentration 50%) is the concentration for which the relative absorbance is 50% of the maximum value.

The mean IC_50_ values [mg nicotine equivalents/mL] derived from the three independent experiments for each test item were compared using analysis of variance, followed by *post hoc* paired comparisons as detailed in Section 2.4.1. *p*-values for comparisons were adjusted using the Bonferroni method to control the family-wise error rate at 0.05. No statistical comparisons were made if the MO test article was deemed noncytotoxic.

## 3 Results

### 3.1 Ames assay

**FIGURE 1:**
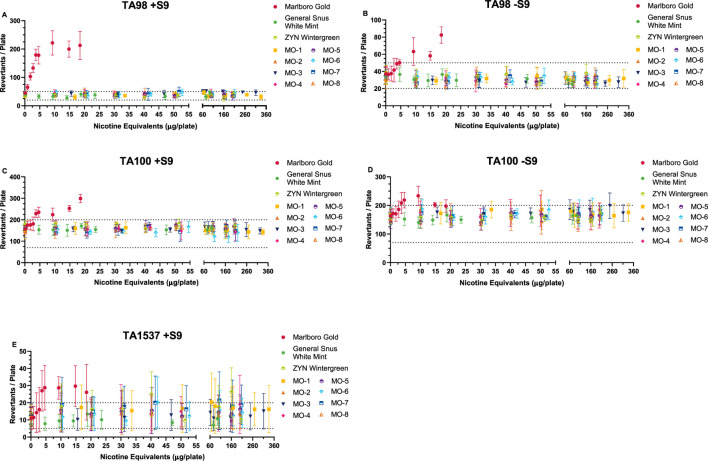
Ames Assay results (preincubation method) in the presence **(A, C, E)** and absence **(B, D)** of metabolic activation (±S9). Methods based on HC T-501 and OECD 471 guidelines. Results from *Salmonella* tester strains TA98 (±S9) **(A, B)**, TA100 (±S9) **(C, D)**, and TA1537 (+S9) **(E)** are displayed. Data not shown for *Salmonella* tester strains TA102 (±S9), TA1535 (±S9), or TA1537 (-S9) because no mutagenic activity was observed from any of these test items. Combustible cigarette (TPM) was mutagenic in the three strains shown, with dose-related increases exceeding the historical spontaneous revertant count range (-- - -). ONP and snus (CAS) test items consistently showed no mutagenic activity in all five tester strains at nicotine equivalent concentrations that were considerably higher than the combustible cigarette. ONP and snus revertant counts consistently fell within the spontaneous revertant historical range for each tester strain. Tested doses are based on the amount of nicotine equivalents (µg/plate) from the different sample preparations (TPM, CAS). Results (mean ± SD) from three (3) independent experiments.

Overt toxicity (as observed by thinning or scarcity of the bacterial lawn or decrease in revertant counts, colony distribution, and density on the plate) was evident at the top concentration(s) tested in all strains and conditions for all study products. Concentration-related, reproducible increases in revertant counts were observed for the comparator cigarette for *S. typhimurium* strains TA98 (±S9), TA100 (±S9), and TA1537 (+S9). These increases were statistically significant (Dunnett’s test, *p* < 0.01) for at least one dose compared to the solvent control and revertant counts exceeding the Poisson 95% confidence interval for the solvent control. Thus, the comparator cigarette met the criteria for a mutagenic response in these strains (Figure 1).

The MO test products and market ONP and snus comparators were negative for mutagenicity across all *S. typhimurium* strains and conditions (±S9) over the dose ranges tested. Therefore, it was concluded that the MO test products are non-mutagenic.

### 3.2 ivMN assay

**FIGURE 2:**
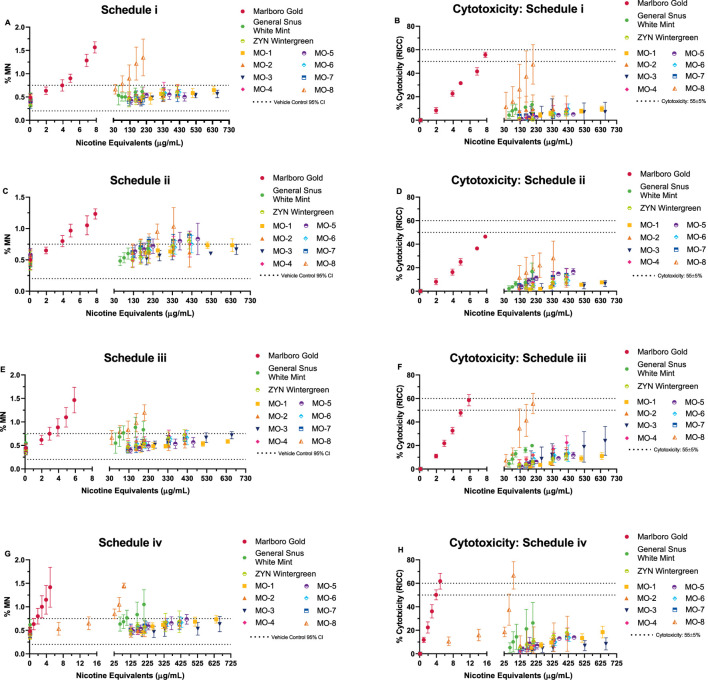
*In vitro* micronucleus (ivMN) assay results **(A, C, E, G)** observed under four exposure schedules (i–iv). ivMN methods (without cytochalasin B) based on HC T-503 and OECD 487 guidelines. Schedule iv, 24 h exposure (-S9) with a 24-h recovery prior to harvesting, referenced from Thorne et al. (2019). Graphs **(B, D, F, H)** display the observed cytotoxicity (based on the relative increase in cell count: RICC) over the same dose range. Combustible cigarette (TPM) displayed genotoxicity in all four exposure schedules, indicated by the dose-related increase in micronuclei (MN) induction. ONP and snus (CAS) exposures resulted in no overall genotoxicity, with the exception of MO-8, which induced MN in all four exposure schedules. ivMN results (mean ± SD) from three (3) independent experiments.

Concentration-related, reproducible increases in the number of induced micronuclei were observed for the comparator cigarette and MO-8 in all treatment schedules; MO-8 was only positive at nicotine equivalent concentrations ranging from 17 to 69-fold higher than the combustible cigarette (Figure 2). The increases were statistically significant (Dunnett’s test, *p* < 0.01) for at least one concentration compared to the vehicle control, and micronucleus counts exceeding the Poisson 95% confidence interval for the vehicle control were observed; therefore, per the criteria for a positive response, the cigarette comparator and the MO-8 test product were deemed genotoxic in all treatment schedules. In contrast, the remaining MO test products and the market ONP and snus comparators were all negative for genotoxicity in all treatment schedules when tested at nicotine equivalent doses up to 42- to 135-fold greater than the combustible cigarette comparator.

Comparison of mean ivMN assay slopes (expressed on nicotine equivalent basis) between the combustible cigarette Marlboro Gold and MO-8 in all four testing schedules were determined to be statistically significant (Table 4) with the slopes of Marlboro Gold indicating a 14–66 times greater induction of MN per nicotine equivalents when compared to MO-8.

**TABLE 4:** ivMN slope analysis of positives.

ivMN Slope analysis	$\frac{\#\text{MN}}{\text{mg}\;\text{Nicotine}\;\text{equivalents}/\text{mL}}$							
	Schedule i		Schedule ii		Schedule iii		Schedule iv	
	CC	MO-8	CC	MO-8	CC	MO-8	CC	MO-8
Slope	1,220.4	42.1	903.5	13.7	1,372.6	30.8	1,762.9	123.5
SD	19.8	21.2	237.8	5.0	272.4	6.1	730.8	18.2
CC vs. MO-8	*p* < 0.001		*p* = 0.003		*p* = 0.001		*p* = 0.018	

### 3.3 NRU assay

**FIGURE 3:**
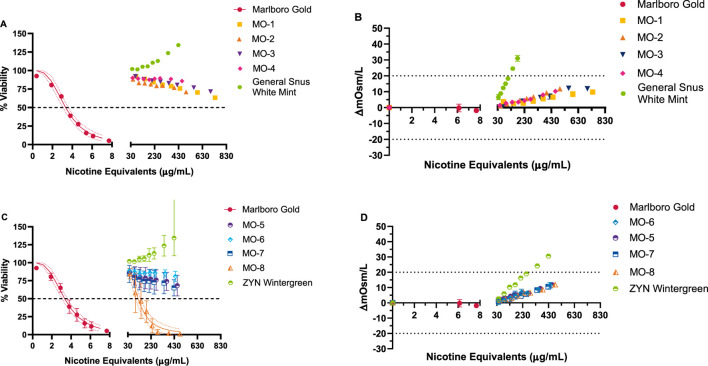
NRU results for Marlboro Gold (TPM) and MO 1–8, ZYN wintergreen, and General Snus white mint (CAS extraction) test items **(A, C)**. Osmolality **(B, D)** was concurrently measured following the exposure, and the osmolality difference from vehicle control was calculated. Dotted lines at ±20% **(C, D)** were used to account for any potential cytotoxic effects induced by osmotic stress. Marlboro Gold TPM and MO-8 CAS extract resulted in cytotoxic responses with a calculated IC_50_ value based on delivery of 3.3 µg nicotine equivalents/mL and 180 µg nicotine equivalents/mL, respectively. No indication of cytotoxicity was observed for Market Snus and MO 1–7 test items up to the maximum deliverable doses. Results (mean ± SD) are from three (3) independent experiments.

The cytotoxicity of the MO test products and the market ONP and snus comparators was tested over a 0.02–0.7 mg nicotine equivalents/mL dose range, whereas the TPM from the comparator cigarette was tested at much lower concentrations between 0.0004 and 0.008 mg nicotine equivalents/mL (Figure 3). Concentration-related, reproducible decreases in neutral red uptake with at least a 50% reduction in neutral red absorbance compared to vehicle control was observed for the comparator cigarette. The cigarette comparator was deemed cytotoxic, with an IC_50_ value of 0.003 mg nicotine equivalents/mL. Among the MO test products, MO-1 to MO-7 did not elicit cytotoxicity. Therefore, IC_50_ values could not be calculated. However, under the test conditions, the MO-8 test product was positive in the NRU assay only at concentrations significantly higher than TPM, with an IC_50_ value of 0.18 mg nicotine equivalents/mL (60-fold higher). The market ONP and snus comparator products were negative for cytotoxicity.

## 4 Discussion

We have assessed *in vitro* toxicological effects of eight modern oral nicotine pouches that varied in nicotine content and flavorings as a part of an overall effort to determine the APPH of these products. The MO products tested in this study were negative or active only at significantly higher nicotine equivalent dose ranges than the combustible cigarettes in the three regulatory toxicological assays. The MO products were not mutagenic, genotoxic, or cytotoxic across various flavors or nicotine content, with the exception of MO-8, which was positive at higher nicotine equivalent doses than the combustible cigarette in the genotoxicity and cytotoxicity assays.

Which factor distinguished MO-8 from the other MO products tested in terms of the positive responses in the ivMN and NRU assays is unknown. The nicotine strength, pouch weight, and pouch size are shared by other MO products. Each extract was prepared at the same concentration (300 mg of MO product/1 mL CAS), and the extractions were over 2 h. This time has been used here and previously (Keyser et al., 2024; Keyser, 2022) with >90% nicotine extraction efficiency (data not shown). This is not unreasonable because >66% nicotine extraction efficiency has been seen at 1 h using a similar approach (East et al., 2021). All of the ingredients used in the formulation of these MO products, including the MO-8 used in this study, have been designated as food ingredients, and/or have been approved by the FDA for direct addition to food for human consumption, and/or have been given the status of GRAS by the FDA (Hall and Oser, 1965). Although it is understood that GRAS status and use as food by the FDA does not directly apply to tobacco products, exposure to these ingredients from the use of MO products occurs through the same route (i.e., oral) through the consumption of food. Therefore, we believe the underlying GRAS determination is relevant to the use of these ingredients in these MO products.

Within the tobacco product risk continuum, combustible cigarettes have been established as products posing the highest health risk to users, with smokeless tobacco products placed at the lower end and nicotine replacement therapy products (NRT), such as nicotine gum, acknowledged as minimal risk products (Gottlieb and Zeller, 2017; Nutt et al., 2014). Although some US moist snuff and Swedish snus products have been authorized by the US FDA as “modified risk” products, they do contain some toxicants, and the FDA requires reporting of some HPHCs for smokeless tobacco products (Food and Drug Administration, 2012b). The ONPs are a new category of products that contain pharmaceutical-grade nicotine, are devoid of other tobacco-derived materials, and could serve as a potentially reduced-risk alternative tobacco product for smokers who switch. A recent review found that ONPs may convey lower risk than cigarettes, with a suggestion for additional studies to further assess population health effects (Grandolfo et al., 2024). Findings reported in our study indicate that the tested MO products could be less harmful alternatives for smokers. Notably, although the MO-8 test product was not mutagenic, it was only positive for genotoxicity and cytotoxicity at significantly higher nicotine equivalent doses than the TPM from cigarette smoke.

With ONPs being a new category of tobacco-nicotine products, test methods for assessing their health effects are under development. Several methods for the generation of test samples from oral products, including the use of CAS extractions, have been reported in the literature (East et al., 2021). Findings from contemporary *in vitro* toxicological assays using extracts of 4–11 mg nicotine/pouch LYFT ONPs prepared using cell culture medium showed that the ONP extracts were minimally cytotoxic and marginally positive only in one (cytotoxicity) among the several endpoints evaluated (East et al., 2021). Phosphate-buffer extracts of 5.8–10.9 mg nicotine/pouch Skruf ONP evaluated with *in vitro* toxicological assays were found to be negative in Ames and ivMN assays (Yu et al., 2022). However, the investigations found that these products were “weakly cytotoxic” in BEAS2B or HepG2 cells, although not reaching an EC_50_ at the top testing concentration of 10 mg/mL. *In vitro* toxicological assessment of CAS extracts of Nordic Spirit ONPs revealed that these products were negative for genotoxicity, mutagenicity, and cytotoxicity at the highest tested dose of 17.14 mg/mL (Miller-Holt et al., 2022). Notably, authors of these prior publications have also employed established methods (such as the Health Canada methods for tobacco product evaluations) for the *in vitro* toxicological assays, similar to those described in this work.

The nicotine content of the MO test articles was mainly 8 mg/pouch, with one test article (MO-4) at 12 mg/pouch, which is higher than that reported for LYFT (East et al., 2021), and comparable to Skruf and Nordic Spirit ONPs (Miller-Holt et al., 2022; Yu et al., 2022). At both nicotine pouch concentrations, the MO test products (with the exception of MO-8) were negative in the *in vitro* toxicological assays. Several flavor variants of the MO test products were assessed, and the flavors were designated per groupings in a previously published flavor wheel (Krüsemann et al., 2019). Although the *in vitro* toxicological assays are described as screening tools (Lauterstein et al., 2020), they constitute an important part of product marketing applications to the FDA (Food and Drug Administration, 2023) and are informative about perturbations in key biological processes in the development of smoking-related diseases (Centers for Disease Control and Prevention et al., 2010a). These screening tools can inform regulators about the cytotoxic, genotoxic, and mutagenic toxicity of tobacco products and be used as part of a weight of evidence for APPH. Thus, the results from the assessments suggest that compared to combustible cigarettes, the MO test products and market ONP and snus comparator products elicit either no or minimal responses in the three regulatory toxicological assays compared to the combustible cigarette comparator, suggesting that these MO test products may be less toxic than combustible cigarettes and beneficial as means of tobacco harm reduction.

A limitation of this study is that we measured only nicotine and did not carry out a detailed chemical analysis of the CAS extracts of MO test products. Several recent reports have indicated that ONPs generally have far fewer and significantly lower levels of toxicants than combustible cigarettes and other tobacco products. For example, a market survey of ONPs determined select HPHC carcinogens, including TSNAs, benzo [a]pyrene, beryllium, cadmium, nitrite, formaldehyde, acetaldehyde, crotanaldehyde, cobalt, lead, nickel, chromium, and selenium are detected in 21 leading brands of ONPs. The overall levels of the HPHCs were found to be at or below the levels observed in traditional STs (Jablonski et al., 2022). Azzopardi et al. chemically characterized four variants of LYFT ONPs, along with three varieties of snus and two NRTs, and reported that the LYFT ONPs and the NRTs contained significantly lower levels of the targeted toxicants than snus (Azzopardi et al., 2022). In that study, the ONPs were generally shown to contain lower levels of HPHCs relative to traditional STs, and the authors suggested that ONPs may be positioned between Swedish snus and NRTs within the tobacco and nicotine product risk continuum (Azzopardi et al., 2022). Although low levels of some HPHC were detectable in ZYN, the comparator ONP used in this study did not contain TSNAs or PAHs (Back et al., 2023).

Another limitation of this work is the effects of these products over time (e.g., days, weeks, years). The ONP class is new, and the long-term (i.e., decades) use of these products in this class is unknown. However, a recent cross-sectional clinical study of the biomarkers of exposure/potential harm to exclusive Velo users (not the same products as used here) showed that several biomarkers linked to the development of lung cancer (NNAL and NNK) and cardiovascular disease (COHb and 11-dTX B2), inflammation (white blood cells), and lung inflammation (FeNO) were significantly lower than in smokers (Azzopardi et al., 2023). Others have shown that switching from Swedish snus to ZYN during a 6-week observational study found a statistically significant decrease in the severity of oral lesions (Alizadehgharib et al., 2022). These reports and others support an overall reduction in harm when cigarette consumers switch to these products rather than continuing to smoke (Grandolfo et al., 2024).

The next steps for this work could investigate the mechanism in which MO-8 was positive in the ivMN and NRU assays as well as use next-generation cell models (i.e., 3D) of the oral cavity, which is how these products will be used. The cytotoxicity of oral tobacco products using a 3D cell model of the buccal region (EpiOral™) has been studied before (Keyser, 2022); however, to the authors’ knowledge, genotoxicity and mutagenicity studies have not been developed for the oral cavity. The development of new approach methods (NAMs) for these endpoints for the oral cavity could improve the detection of the mutagenicity, genotoxicity, and cytotoxicity of oral products.

In summary, the MO test products (1–7) assessed in this study were negative for mutagenicity, genotoxicity, and cytotoxicity. The MO-8 test product was not mutagenic; however, it was positive in the ivMN and NRU assays when tested at doses that were several-fold higher in terms of nicotine equivalents than combustible cigarettes. Overall, the findings reported herein support the hypothesis that ONPs are potentially less toxic than combustible cigarette products and provide a lower-risk alternative to current combustible cigarette smokers.

## Data Availability

The original contributions presented in the study are included in the article/supplementary material; further inquiries can be directed to the corresponding author.
